# Efficacy of Aflibercept 8 mg in Pretreated Age-Related Macular Degeneration

**DOI:** 10.3390/jcm14144900

**Published:** 2025-07-10

**Authors:** Christiane Palm, Sandrine Anne Zweifel, Felix Gabathuler, Mariano Cozzi, Katrin Fasler

**Affiliations:** 1 Department of Ophthalmology, University Hospital Zurich, University of Zurich, 8091 Zurich, Switzerlandmariano.cozzi@usz.ch (M.C.); katrin.fasler@usz.ch (K.F.); 2 Eye Clinic, Department of Biomedical and Clinical Sciences, Luigi Sacco Hospital, University of Milan, 20157 Milan, Italy

**Keywords:** aflibercept 8 mg, age-related macular degeneration, intra-vitreal injections

## Abstract

This study aims to evaluate the real-world efficacy and safety of aflibercept 8 mg intravitreal injections (IVTs) in pretreated patients with neovascular age-related macular degeneration (nAMD) throughout the first three IVTs. **Background**: Established anti-vascular-endothelial-growth-factor (anti-VEGF) therapies positively impact the progression of nAMD but require frequent administration, thus burdening patients and the healthcare system. Pivotal trials of the recently approved aflibercept 8 mg have demonstrated extended dosing intervals with comparable safety to standard treatments. However, real-world data is still scarce. **Methods**: A retrospective, single-center single-arm analysis was conducted on 22 eyes from 18 pretreated nAMD patients. Eyes were switched from other anti-VEGF agents to aflibercept 8 mg injections continuing a treat-and-extend regimen (no loading dose after switching). Treatment intervals and structural (central subfield thickness (CST); disease activity) and functional (best corrected visual acuity (BCVA)) outcomes were assessed at baseline (date of first aflibercept 8 mg injection) and at follow-up examinations until follow-up 3. Safety data, including intraocular pressure changes, were recorded. **Results**: Over a median follow-up of 16.6 weeks (IQR 15.1–27.0), patients switched to aflibercept 8 mg showed prolonged intervals between injections (5.5 weeks vs. 7 weeks, *p* < 0.001, Wilcoxon signed-rank test), reduced disease activity, stable CST, and stable BCVA. One patient experienced transient intraocular pressure elevation, which resolved without intervention. No other adverse events were observed. **Conclusions**: Treatment with aflibercept 8 mg appears to provide effective disease control with prolonged treatment intervals in switched nAMD patients in routine clinical practice. These findings further indicate the potential for reducing treatment burden.

## 1. Introduction

Age-related macular degeneration (AMD) accounts for a significant proportion of vision impairment globally, with the number of affected people being predicted to reach 288 million by 2040 [1,2]. Neovascular AMD (nAMD) is critically driven by pathological vascular-endothelial-growth-factor (VEGF) signaling [3], leading to development of macular neovascular membranes (MNVs) and vision-threatening exudation. The role of intravitreal anti-VEGF injections (IVTs) has evolved in the last decade and resulted in improvements in functional as well as anatomical outcomes for nAMD, which was investigated extensively in randomized trials as well as under real-world circumstances [4,5,6,7]. However, the necessity for the frequent administration of the treatment places a significant burden on patients and public healthcare systems [8,9], which is why efforts have been made to improve the durability of the treatment effect and thus reduce treatment frequency.

Aflibercept, a commonly used and well-studied anti-VEGF agent, was approved at the increased dosage of 8 mg by the FDA in August 2023 and the European Commission in January 2024 following pivotal studies [10,11]. The CANDELA study (phase 2) demonstrated greater fluid resolution potential and low side effect rates compared to 2 mg of the drug [11]. In the PULSAR phase 3 trial, the 8 mg dose was demonstrated to be comparably safe, while showing a numerically shorter median time to fluid-free central subfield as well as an increased improvement of best corrected visual acuity (BCVA) with extended treatment intervals [10]. Matsumoto et al. described first real-world data of treatment-naive and pretreated AMD patients in Japan 4 weeks after their first aflibercept 8 mg injection [12]. However, data on the safety and efficacy of the newly approved 8 mg dosage in pretreated patients with a longer follow-up under real-world circumstances has yet to be published.

This study reports the first real-world data on the safety and efficacy of switching pretreated patients with nAMD to three injections of aflibercept 8 mg. In accordance with routine clinical practice, our aim was to evaluate the treatment outcome of a treat-and-extend regimen without the traditional monthly loading phase.

## 2. Materials and Methods

### 2.1. Ethics

Approval of the Ethics Committee of the Canton of Zurich, Switzerland, was obtained with Project Nr PB_2016_00264. This study conforms to the ethical standards established in the 1964 Declaration of Helsinki and its later amendments.

### 2.2. Study Design and Protocol

This study reports safety and efficacy outcomes after 3 injections of aflibercept 8 mg as part of a retrospective, single-center, single-arm analysis at the Department of Ophthalmology at the University Hospital Zurich (USZ), Switzerland.

**Figure 1:**
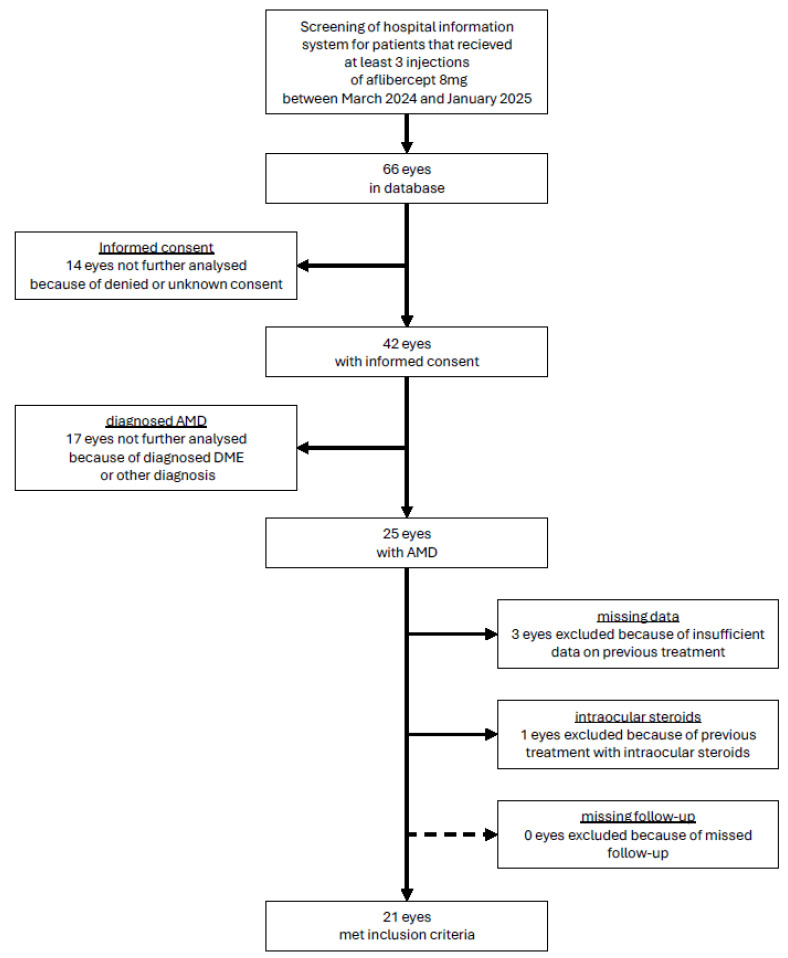
Flow chart of inclusion and exclusion of eyes.

Included were consenting and pretreated patients diagnosed with age-related macular degeneration who received three injections of aflibercept 8 mg at USZ between March 2024 and January 2025. Exclusion criteria were missed completion of three follow-up examinations at USZ between March 2024 and January 2025, incomplete data regarding previous treatment, as well as concurrent treatment with intraocular steroids. A visualization of the in- and exclusion criteria is shown in Figure 1.

### 2.3. Data Collection

**Figure 2:**
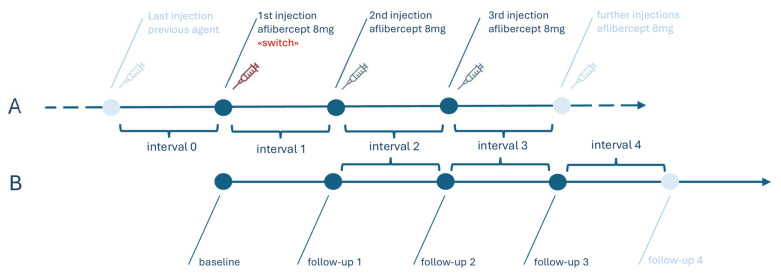
Visualization of the set-up of injections, follow-up examinations, and intervals. Timeline (**A**) represents the performed intravitreal injections and timeline (**B**) the follow-up examinations as well as the treatment intervals between them.

The functional and anatomical data of the first injection date with aflibercept 8 mg was defined as “baseline.” The follow-up examinations were conducted on the date of the following dose of aflibercept 8 mg, preceding the injection (Figure 2). Participants continued aflibercept 8 mg injections with the treatment interval that was established with their latest anti-VEGF agent, without a loading phase (i.e., switch “on the fly”). IVT with aflibercept 8 mg was continued in a treat-and-extend regimen (treatment interval extension of 2 weeks if inactive, treatment interval shortening of one week if active). The treatment interval before switching was defined as interval 0. The last interval in our study, which was set as the treatment interval determined at follow-up examination 3, was defined as interval 4. The last follow-up examination included in this study (follow-up 3) took place on the date of the planned fourth injection, with the overall time span between the baseline and the follow-up 3 covering at least twelve weeks.

Clinical patient data, including safety markers and disease activity, was continuously monitored and documented. For this paper’s analysis, the documentation was sourced from the clinic’s hospital information system KISIM (CISTEC AG, Zurich, Switzerland). Corrected visual acuity (BCVA) was evaluated with auto refraction (NIDEK NT-530/510, Nidek Company, Ltd., Hirioshi-cho, Gamagori, Aichi, Japan) and collected according to the early treatment of diabetic retinopathy study (ETDRS) letter score. Intraocular pressure (IOP) was measured by air puff tonometry (Nidek Company, Ltd., Hirioshi-cho, Japan) or Goldman applanation tonometry (Haag-Streit Group, Köniz, Switzerland).

Clinical data on safety, including anterior chamber (AC) cells, vitreous cells, and retinal vessel status, was assessed at the slit lamp.

Retinal scans were performed by spectral-domain optical coherence tomography (SD-OCT) (Spectralis, Heidelberg Engineering, Heidelberg, Germany).

All structural OCT scans were reviewed for image quality, ETDRS grid foveal centration, and segmentation boundaries, with manual corrections performed if necessary.

Central subfield thickness (CST) and subretinal fluid (SRF) were measured in micrometers as a structural-anatomical correlate for treatment efficacy. All measurements were performed twice by separate reviewers and any discrepancies >3 μm were discussed until a consensus was reached.

### 2.4. Outcome Measures

We defined the extension of treatment intervals after the switch to aflibercept 8 mg as a primary outcome. Intervals between injections were recorded in weeks to measure the duration of the treatment effect. Clinical disease activity, safety, and anatomical/functional response were defined as secondary outcomes. CST was used as the common anatomical surrogate measures for structural efficacy. Disease activity (defined as presence of SRF, intraretinal fluid (IRF), or hemorrhage) was used as surrogate of clinical treatment response. BCVA was measured as the functional outcome.

### 2.5. Statistical Analysis

All statistical analyses were conducted using R software version 4.1.2 (R Project—The R Foundation for Statistical Computing, Vienna, Austria). For quantitative measures, normal distribution was tested using the Shapiro–Wilk test. For normally distributed variables, mean and standard deviation were presented. For non-normally distributed variables, median and interquartile range (IQR; 25th–75th interquartile range) were presented, and for discrete values, numbers and percentages were presented.

The ANOVA test for repeated measurements was conducted for normally distributed variables, including CST. A Friedman rank sum test was conducted to evaluate changes across follow-up visits for the treatment interval and BCVA. This non-parametric test was chosen due to the repeated-measures design and the non-normal distribution of the data. In cases where the Friedman test indicated statistically significant differences, Wilcoxon signed-rank tests were performed for pairwise comparisons to identify specific time points showing significant change. All statistical tests were two-sided, and a *p*-value < 0.05 was considered statistically significant.

## 3. Results

### 3.1. Demographics and Clinical Characteristics

This analysis includes 22 eyes from 18 patients with a mean age of 77.1 years (SD 7.6). The 22 eyes were pretreated with a median of 60 (IQR 16.0–77.8) injections prior to switching to aflibercept 8 mg. The most commonly used agent before switching was aflibercept 2 mg (13/22). Patients had been pretreated with a median of two (IQR 1–4) different anti-VEGF agents before the switch to aflibercept 8 mg. The previously used agents were aflibercept 2 mg, ranibizumab, brolucizumab, and faricimab. The median reached treatment interval between injections before switching to aflibercept 8 mg was 5.5 weeks (IQR 5.0–8.0). The median time span covered from baseline to last follow-up examination was 16.64 weeks (IQR 15.14–27.00). The demographics of the 18 patients who completed the follow-up are shown in Table 1.

### 3.2. Treatment Interval and Efficacy

**Figure 3:**
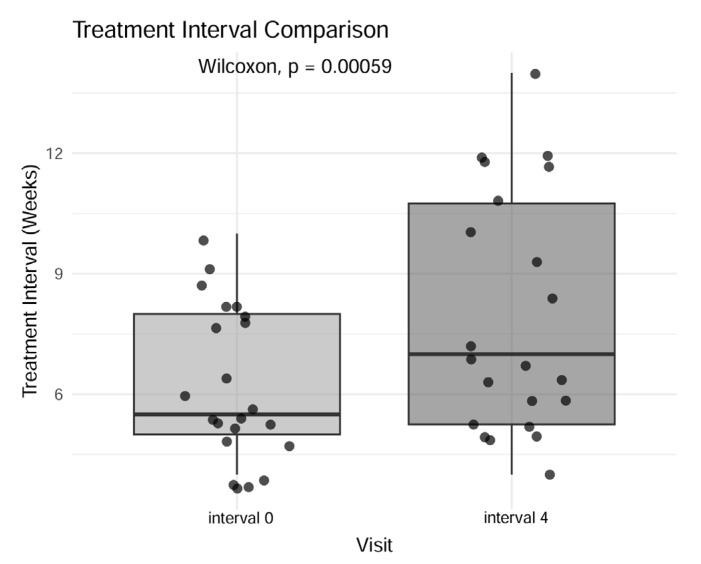
Boxplot of treatment intervals between baseline treatment interval (interval 0) and the last treatment interval (interval 4).

We found a statistically significant difference in treatment intervals across different visits (Friedman rank sum test, *p* < 0.001). After pairwise comparison, the only significant increased treatment interval was achieved between interval 0 (before switch) and interval 4 (Figure 3), with a median increase of 1.5 weeks between consecutive injections of aflibercept 8 mg (Wilcoxon signed-rank test, *p* < 0.001).

**Figure 4:**
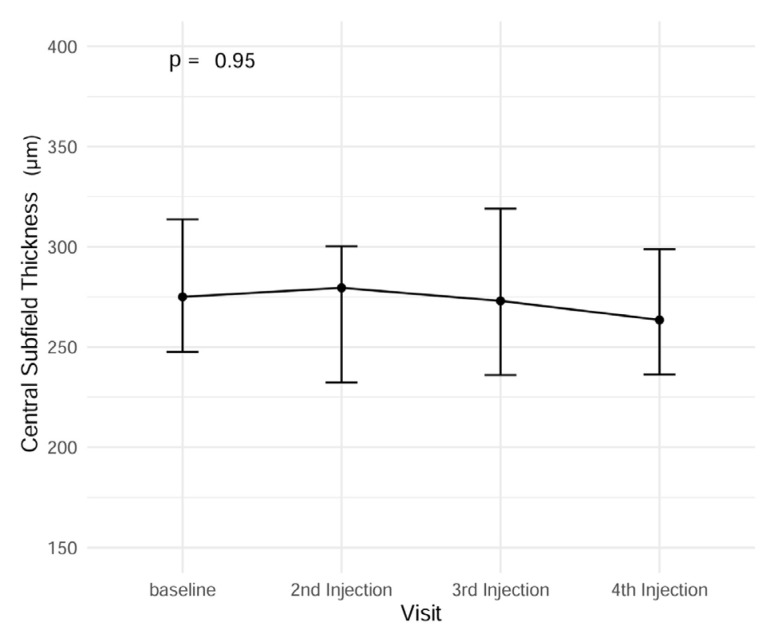
Central subfield thickness (CST) during the follow-up examinations. Mean baseline CST (SD) = 280.0 (53.4) µm; mean follow-up 3 CST = 271.4 (55.8) µm.

**Figure A1:**
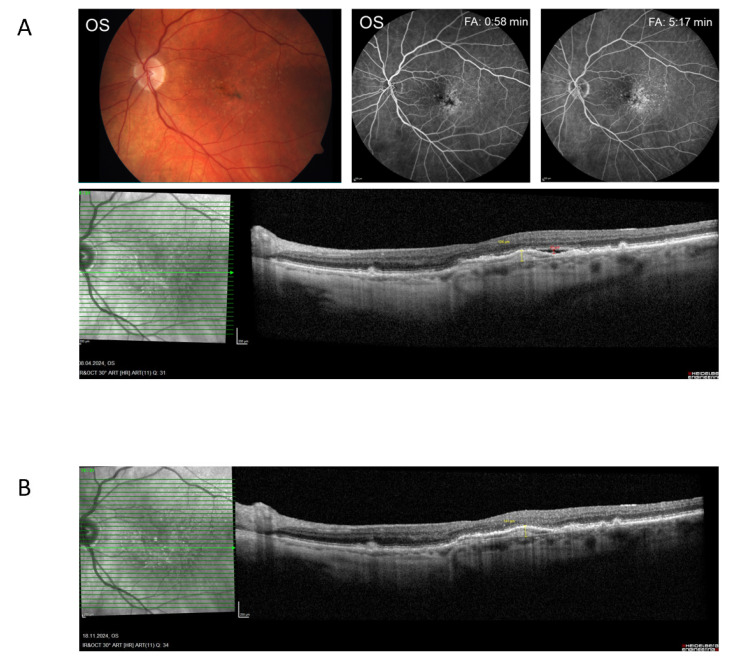
Exemplary case of nvAMD in the left eye with active type 1 MNV. (**A**) Top row shows fundus photography (left) and angiography images (middle and right) at initial diagnosis of type 1 MNV with drusen, pigment epithelial detachment (PED)/changes, and staining of fibrovascular PED. Bottom row shows infrared and OCT images at baseline with fibrovascular PED, drusen, and subretinal fluid (classified as disease activity). (**B**) Infrared and OCT image at follow-up 3 showing fibrovascular PED without any subretinal fluid (classified as no disease activity).

While CST did decrease over the course of observation, this difference was not proven to be significant (ANOVA, *p* = 0.95) throughout the observation period (Figure 4). However, the documented disease activity during clinical consultations was significantly reduced over the observation time. At baseline, 16/22 eyes were classified as showing signs of disease activity, while at the last follow-up, only 6 eyes still showed signs of disease activity. This translates to a reduction in disease activity of 45.5% (Figure 4, Appendix A Figure A1).

**Figure 5:**
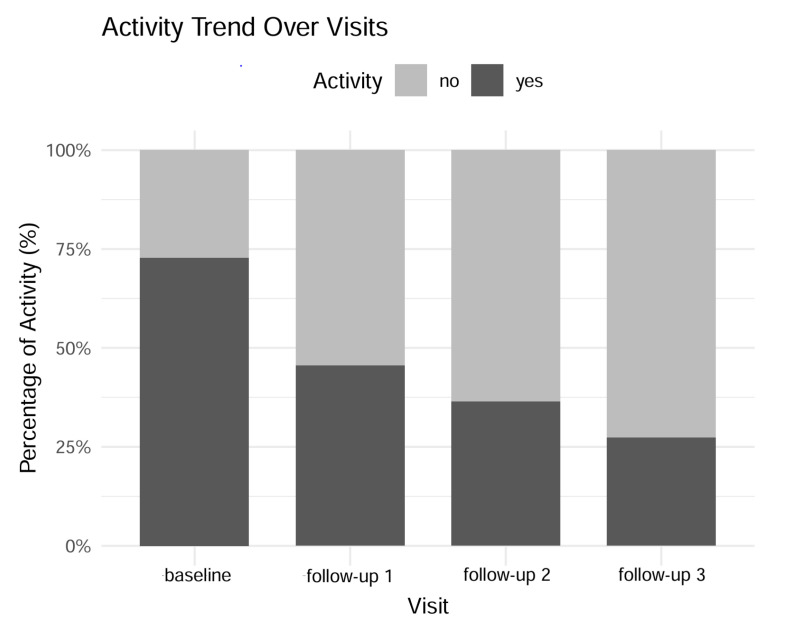
Percentage of eyes with clinical signs of activity throughout the follow-up examinations.

BCVA proved to remain stable (Figure 5) over three follow-ups, with no significant change (Friedman rank sum test, *p* = 0.228). Patients exhibited a median ETDRS letters score of 76 (IQR 70.8–79.0) at baseline, while the median at third follow-up was 74.5 (IQR 68.5–79).

### 3.3. Safety

The safety profile was monitored at the regularly scheduled follow-up appointments. While the mean IOP remained stable throughout the follow-up appointments (*p* = 0.91), episodes of temporary symptomatic IOP elevation post-injection with aflibercept 8 mg were documented in one patient. This patient developed symptomatic IOP elevation (impairment of vision below count fingers with hard bulbus on palpation) immediately after their second and third injections. Before the third injection, peroral acetazolamide 250 mg was administered 30 min before IVT; however, the patient still developed a temporary symptomatic IOP elevation. In all instances, however, the IOP normalized spontaneously or after ocular massage without the need for paracentesis or other medical intervention. No anterior chamber cells, flare, vitreous cells, or vasculitis were detected at any assessment point in any eye.

## 4. Discussion

Our study shows that pretreated nAMD eyes switched to aflibercept 8 mg by IVT without a loading phase demonstrated extended treatment intervals and a reduction of disease activity, with stable BCVA and no new safety signals in routine clinical practice.

Patients included in this analysis had a median of 60.0 previous injections and a treatment interval of 5.5 weeks prior to the switch to aflibercept 8 mg. After the third follow-up, a significant extension of treatment interval to a median of 7 weeks was reached, while simultaneously reducing the percentage of eyes with disease activity by 45.5%. Since aflibercept 8 mg was administered to treatment-naive eyes in the pivotal CANDELA and PULSAR studies, eyes were started on three monthly loading doses before continuing with extended intervals. In the CANDELA trial, patients then received doses at weeks 20 and 32, with additional doses applied at the discretion of the investigators, adding up to a median of 5.8 administered injections per eye by week 44. In the pivotal PULSAR study, Lanzetta et al. report that 83% of eyes maintained at least a 12-week treatment interval by week 48. Compared to the results of these pivotal trials, the significant treatment interval extension in our “high-need” cohort (median of 60 previous IVTs, median treatment interval 5.5 weeks at baseline) indicates a translatable benefit of aflibercept 8 mg beyond highly controlled clinical trial settings.

In terms of structural response, while a CST reduction of 8.6 µm was not significant, we noted a decreased rate of disease activity over the course of observation. In the CANDELA trial, an increase in the percentage of patients with a fluid-free central subfield of 11.6% when comparing the aflibercept 8 mg to the aflibercept 2 mg treatment arms at week 44 was described. Additionally, the aflibercept 8 mg treatment arm reached a higher reduction of CRT at week 44, while both treatment arms received a mean of 5.8 injections in.

In the pivotal PULSAR study, in the 8 mg/12-weeks and 8 mg/16-weeks groups there were 71% and 67% of patients without fluid in the central retinal subfield at week 48, respectively, compared to 59% in the 2 mg/8-weeks treatment arm. CST was reduced by 141.9 µm and 147.1 µm in the 8 mg/12-weeks and 8 mg/16-weeks groups, respectively, compared to 126.3 µm in the 2 mg/8-weeks group. Matsumoto et al. reported a significant decrease in foveal thickness and central choroidal thickness at 4 weeks after the first injection but provided no further data from a longer follow-up. While not comparable to this data, the reduction in disease activity in our cohort is not necessarily to be expected but supports the superior efficacy of aflibercept 8 mg.

Regarding functional outcomes, our patients did not show a significant BCVA change after three aflibercept 8 mg IVTs. In a heavily pretreated cohort, functional improvement is not to be expected, and previous studies on switching to other anti-VEGF agents have shown the same results [13]. Matsumoto et al. provided the only other real-world-evidence of aflibercept 8 mg application, observing 18 treatment-naïve and 17 pretreated eyes four weeks after their first aflibercept 8 mg IVT and reporting significant improvement in BCVA at the follow-up examination; however, the authors provided no further data regarding a longer follow-up [12]. With 50% treatment-naïve eyes, this study is not comparable to our patient cohort.

Apart from one temporary symptomatic IOP elevation in one patient, no other/new safety signal was found in our study. Since this patient had no history of post-injectional pressure elevation with their previous anti-VEGF agents, the injected volume (0.07 mL in aflibercept 8 mg compared to 0.05 mL in aflibercept 2 mg) might have played a role in the pressure rise in this patient. In the CANDELA and PULSAR trials, the incidence of treatment emergent adverse events was similar between aflibercept 8 mg and aflibercept 2 mg. In the CANDELA trial, no vascular occlusive events but one case of iritis occurred. In the PULSAR trial, there were few intraocular inflammation events, with the most common adverse events being reduced visual acuity, cataracts, and retinal hemorrhage.

In recent research, Paris et al. compared post-injectional IOP changes between faricimab, aflibercept 2 mg, and aflibercept 8 mg, and observed no difference between aflibercept 2 mg and aflibercept 8 mg in the prevalence or degree of the pressure elevation [14]. Matsumoto et al. [12] reported non-infectious IOI in 3/35 eyes at 4 weeks. Hoffmann et al. reported a case series of eight patients who developed sterile IOI after aflibercept 8 mg IVT [15].

The limitations of this analysis include its retrospective nature and the relatively small study cohort. The inclusion of only pretreated patients was expected to lead to inferior results regarding the structural and functional results compared to studies including treatment-naïve patients. However, the analysis of the treatment effect in this group is just as relevant in clinical practice, and we believe that focused research in this subgroup supports future clinical decision making. We were thus willing to accept the selection bias of exclusively analyzing pretreated patients in order to adapt our research to the actual real-world context of treatment implementation. The subsequent advanced treatment history and the timing of the switch resulting in the observed improvement might be partly explained by a regression to the mean, especially since this analysis was conducted without a control group.

We see the real-world setting of this analysis and specifically the focus on a cohort of extensively pretreated patients as a key strength of this analysis. Despite the expectation of a potentially limited treatment effect in this cohort, we observed positive effects across all evaluated outcome measures. Practical information on the switch to aflibercept 8 mg without a loading phase in pretreated patients, reflecting the population frequently encountered in clinical practice, adds translational value to these findings.

## 5. Conclusions

In conclusion, this paper shows real-world evidence that supports the use of aflibercept 8 mg as a potent anti-VEGF option to extend treatment intervals while maintaining low disease activity in pretreated nAMD patients. The significant reduction in disease activity, stable BCVA with extended intervals, and no new safety concerns were achieved in a cohort of patients with advanced treatment history. Additionally, aflibercept 8 mg was administered without the need for a loading phase, suggesting that aflibercept 8 mg can be integrated into ongoing treatment plans. However, studies with larger cohorts and a prospective study design, as well as a longer follow-up are needed to further validate the efficiency and safety of aflibercept 8 mg under real-world conditions.

## Figures and Tables

**Table 1:** Characteristics at baseline.

Patients, n	18
Female, n (%)	8 (44.4%)
Age in years, mean (SD)	77.1 (SD 7.6)
Eyes, n	22
last treatment interval before switch in weeks, median (IQR)	5.5 (5.0–8.0)
previous number of IVTs, median (IQR)	60.0 (16.0–77.8)
pretreated, n (%)	22 (100%)
previously used no. of different agents, median (min-max)	2 (1–4)
last used anti-VEGF agent before switch, n of eyes (%)	
Aflibercept 2 mg	13 (59.1%)
Ranibizumab	3 (13.6%)
Faricimab	6 (27.3%)
Baseline BCVA in ETDRS letters, median (IQR)	76 (70.8–79.0)
IOP in mmHg, mean (SD)	15.0 (3.8)
CST in micrometers, mean (SD)	280.0 (53.4)
SRF in micrometers, median (IQR)	36.0 (0.0–81.5)
MNV Type n (%)	
type 1	19 (86.4%)
mixed type (1&2)	3 (13.6%)

## Data Availability

The original contributions presented in this study are included in the article. Further inquiries can be directed to the corresponding author.

## Abbreviations

The following abbreviations are used in this manuscript:

AMD	Age-related Macular Degeneration
nAMD	Neovascular Age-related Macular Degeneration
anti-VEGF	Anti-Vascular Endothelial Growth Factor
IVT	Intravitreal Injection
BCVA	Best-Corrected Visual Acuity
CST	Central Subfield Thickness
SRF	Subretinal Fluid
IRF	Intraretinal Fluid
ETDRS	Early Treatment Diabetic Retinopathy Study
IOP	Intraocular Pressure
SD	Standard Deviation
IQR	Interquartile Range
OCT	Optical Coherence Tomography
SD-OCT	Spectral Domain Optical Coherence Tomography
MNV	Macular Neovascularization
AC	Anterior Chamber
CRT	Central Retinal Thickness
USZ	University Hospital Zurich
